# Diabetes

**Published:** 1891-05

**Authors:** 


Diabetes; Its Causes, Symptoms, and Treatment. By Charles W. Purdy, M. D., Queen's University. Philadelphia: 1231 Filbert Street, F. A. Davis, Publisher. 1890. Price, $1.25 net.

This volume of 184 pages constitutes No. 8 of the "Physicians' and Students' Ready Reference Series," several previous numbers of which we have from time to time noticed.

The object of the volume, as stated in the preface, is to furnish the physician and student with the present status of our knowledge on the subject of diabetes in such practical and concise form as shall best meet the daily requirements of practice.

Accordingly diabetes mellitus is defined, on page 19, to be a "disease characterized by a perverted elaboration in the economy of the food products whereby chiefly, though not exclusively, the carbo-hydrates become converted into sugar, and the efforts of the system to eliminate the latter give rise to certain symptoms and disturbances." "Our present knowledge of physiological chemistry renders it more than probable that this disturbance is seated chiefly in the liver, and for the last fifty years the most earnest efforts have been put forth in attempts to unravel the nature of this morbid process."

For the benefit of such of our readers as may not have the time to peruse this excellent book, and the better to show its character, we make the following selections from the text, page 19:

"Bernard laid the foundation for subsequent research by demonstrating that one of the functions of the liver in health is the formation and storing up of gylcogen or animal dextrine—a substance chemically identical with starch. Bernard showed that when an animal is recently killed and the liver removed and placed in a warm place, it soon becomes charged with sugar by the conversion of part of this glycogen into glucose. If, next, all the sugar be washed out of the liver by means of a stream of water, and the organ be permitted again to remain in a warm place for twenty-four hours, it becomes abundantly charged with sugar. This may be repeated again and again until finally all the glycogen contained in the liver is converted into sugar. Since the sugar obtained from glycogen or animal dextrine in the liver is identical in all respects with the glucose found in diabetic urine, it cannot be doubted that the source of diabetic urine is the liver."

Page 20: "It has just been stated that glycogen is chemically identical with starch. They are both convertible into glucose by contact with saliva, pancreatic juice, or diastase. They possess one important difference, however, viz., glycogen is converted into glucose by contact with arterial blood, while starch remains unchanged by the latter. The blood, therefore, contains a peculiar ferment capable of converting animal dextrine into sugar; as yet this ferment has not been isolated."

This rather copious extract gives the reader a glimpse of the probable origin of the sugar that appears in the urine. The remainder of the argument we have not space to present here and must refer all interested inquirers to the book itself. The etiology, morbid anatomy, and symptomatology are given in detail, concisely and yet satisfactorily, and then follows the treatment.

In this regard, the main reliance is upon selected food. At page 81 the author says: "Until future investigation shall have revealed some agency through which we are able to check the excessive formation of sugar in the liver, our chief resource against the disease must consist in withholding from the system that which it is capable of converting into sugar, and in supplying that which it is capable of assimilating as nourishment." Accordingly, all foods containing starch and sugar in any form are withheld. This is especially true of bread. Alcoholic beverages are not withheld unless they contain sugar. A complete list of wines, with the quantities of sugar they respectively contain, is given, and full statements of foods allowed and forbidden are added. Thus the reader can, in a very moderate compass, get a very clear knowledge of all he wishes to know concerning this disease. This is a characteristic of the whole series of books to which this one belongs.

